# Obesity Mediates Apoptosis and Extracellular Matrix Metabolic Imbalances via MAPK Pathway Activation in Intervertebral Disk Degeneration

**DOI:** 10.3389/fphys.2019.01284

**Published:** 2019-10-10

**Authors:** Xuyang Zhang, Jian Chen, Bao Huang, Jiasheng Wang, Zhi Shan, Junhui Liu, Yilei Chen, Shengyun Li, Shunwu Fan, Fengdong Zhao

**Affiliations:** ^1^Department of Orthopaedics, Sir Run Run Shaw Hospital, School of Medicine, Zhejiang University, Hangzhou, China; ^2^Key Laboratory of Musculoskeletal System Degeneration and Regeneration Translational Research of Zhejiang Province, Hangzhou, China

**Keywords:** obesity, apoptosis, extracellular matrix, MAPK pathway, intervertebral disk degeneration

## Abstract

Obesity may promote intervertebral disc degeneration (IDD) by non-mechanical means, by influencing levels of free fatty acids which could impair cell metabolism. This study aims to establish metabolic factors in obesity-related IDD independent of mechanical loading. In clinical study, we retrospectively reviewed 128 volunteers (73 males, 55 females, aged 29–88 years) and compared their grades of disk degeneration with obesity-related factors such as body weight, BMI, and serum lipid levels. Clinically, the IDD group showed increased age, BMI and serum triglyceride. Triglyceride was a significant risk factor for IDD even after correction for BMI and age (P = 0.007). In obesity animal model, rats were fed a high-fat diet (HFD) in order to study its effects on disk metabolism and apoptosis. HFD rats had significantly higher serum levels of lipids, including triglyceride and non-esterified fatty acid, and showed significantly decreased markers of anabolism, increased catabolism and apoptosis in disk. Finally, rat nucleus pulposus (NP) cells were stimulated *in vitro* with a fatty acid (palmitic acid, PA) to gauge its effects on cell metabolism and apoptosis. Cell culture studies showed that NP cells exposed to PA showed increased apoptosis for activation of caspase 3, 7, 9, and PARP, which was primarily via the MAPK signal pathway, especially ERK pathway. In conclusion, hypertriglyceridemia can lead to IDD, independently of age and BMI. Hypertriglyceridemia appears to mediate disk cell apoptosis and matrix catabolism primarily via the ERK pathway.

## Introduction

Low back pain (LBP) can be a chronic disorder that severely impairs quality of life (Waddell and Burton, 2001; German and Foley, 2005). The overall prevalence of LBP in the population is 15%∼30%, of which more than 40% is attributable to intervertebral disc degeneration (IDD) (McBeth and Jones, 2007). The etiology of IDD is complicated, but appears to involve inflammation, excessive mechanical loading, genetic inheritance, and an impaired nutrient supply (Inoue and Espinoza Orias, 2011; Chen et al., 2013; Weiler, 2013; Gologorsky and Chi, 2014; Peng and Lv, 2015).

Obesity is a chronic disease characterized by excessive body fat accumulation, (Melo et al., 2014) with body mass index (BMI) ≥ 28 according to Chinese diagnostic criteria (Zhu et al., 2017). It can affect whole body metabolism and lead to a number of health problems such as high cholesterol, diabetes, cardiovascular disease, arthritis, and tumors (Wang et al., 2006; Yamamoto et al., 2012). Obesity affects more than 2/3 of people in the United States (Yang and Colditz, 2015).

Increasing clinical evidence shows that obesity is closely related to the development of IDD. In adolescent patients, BMI is significantly related to IDD, and IDD is more severe in overweight and obese patients compared with those of normal weight (Samartzis et al., 2011). A single nucleotide polymorphism (SNP) analysis study of fat mass and obesity-associated gene (FTO gene) showed a significant correlation between SNP RS 11076008 loci G/G genotype and IDD in the Han population, which suggests that obesity may be an important factor inducing IDD (Sheng et al., 2017). Abdominal obesity (measured by waist circumference, abdominal fat thickness, and ventral subcutaneous thickness) is positively related with Pfirrmann grade of disk degeneration in young adults (Takatalo et al., 2013), and a comprehensive meta-analysis by Xu et al. (2015b) showed that obesity is one of the highest risk factors for IDD. Obesity could possibly initiate IDD by increasing physical loading of the lumbar spine (Adams and Roughley, 2006) but metabolic influences may also be involved.

Most obese patients have abnormally high blood lipid levels (Chang et al., 2015), and this “Hypertriglyceridemia” can play an important intermediary role in various obesity complications (Kratz et al., 2013; Chang et al., 2015). Immunocytes treated with fatty acids can activate inflammatory signaling pathways such as TLRs and NF-κB, promote the expression and secretion of inflammatory factors such as IL-1β and IL-6, and also activate macrophages to promote inflammatory responses (Fritsche, 2015; Alvarez-Curto and Milligan, 2016). High fat in the blood can induce apoptosis by oxidative stress and endoplasmic reticulum stress, and may be involved in the regulation of MAPK, PI3K and other signaling pathways in liver cells, mesangial cells, and pancreatic beta-cells (Yao et al., 2015; Sramek et al., 2016; Wang et al., 2016). High fat diet treatment significantly upregulated the expression of BMP-2, Msx-2 and osteopontin in mice vascular smooth muscle cells, and promoted their differentiation into osteoblast-like cells, in a process that involved upregulation of expression and activation of the NF-κB signaling pathway (Kageyama et al., 2013). Therefore, the influence of Hypertriglyceridemia on obesity complications appears to be related to increased inflammatory responses, and cell degeneration or apoptosis. However, the effects of hypertriglyceridemia on intervertebral disk cells are unknown, and it remains unclear whether it is increased mechanical loading or hypertriglyceridemia that most influences IDD in obese patients.

The aims of the present investigation are to (a) clarify associations between hypertriglyceridemia and IDD in clinical cases, (b) analyze blood lipid indices that are related to IDD in a high-fat rat model of obesity, and (c) explore specific mechanisms through which fatty acids fat can influence intervertebral disk cell survival and metabolism *in vitro*. This study could further deepen our understanding of IDD, and provide a new theoretical basis for its prevention and treatment.

## Materials and Methods

The clinical study was approved by the ethics review committee of Sir Run Run Shaw Hospital. Volunteers agreed to our study by signing informed consent forms. All animal procedures were approved by the Animal Care and Use Committee at Zhejiang University. Each experiments were performed at least three times.

### Clinical Study

We retrospectively reviewed 128 volunteers (73 males and 55 females) who had received Magnetic Resonance Imaging (MRI) scanning between January 2014 and December 2016. Volunteers were excluded if their radiology and medical history revealed a tumor, vertebral deformity or fracture, tuberculosis, smoking, drinking, diabetes or other diseases affecting blood lipids. Clinical data was collected including: height, weight, body mass index (BMI), total blood cholesterol (TC), triglyceride (TG), high density lipoprotein (HDL), low density lipoprotein (LDL), very low density lipoprotein (VLDL), and apolipoprotein A (Apo A). Disk degeneration was classified from MRI according to Pfirrmann grades (Urrutia et al., 2016) by an experienced radiologist and by a senior orthopedic surgeon, who were blinded to other information. The average grade value for L1-L2, L2-L3, L3-L4, L4-L5 and L5-S1 was calculated for each volunteer, and volunteers were then assigned to the “disk degeneration” group (average Pfirrmann grade ≥ 3.5) or “non-degeneration” group. Finally, volunteers were sorted according to their triglyceride reading into a “high fat” group (TG > 1.7 mmol/L) and a “low fat” group.

#### High-Fat Diet Rat Model of Obesity

Twenty male Sprague–Dawley rats (10 weeks old, average weight 200 g) were housed in the Zhejiang University animal experiment center SPF grade with sufficient food and water. Two weeks later, 10 rats were randomly separated into a sub-group which were given a high-fat diet (D12451, New Brunswick, NJ, United States). This feed contained 45% of its calories as fat (4.7 kcal/g), which contained 24 gm% fat, 41 gm% carbohydrate and 24 gm% protein (Yokono et al., 2014). The remaining 10 rats were given a normal diet (Harlan Teklad, Madison, WI, United States, feed #7001, 3.0 kcal/g), which contained only 4.25% of its calories as fat. Body weight and body length of rats were recorded every week, as well as their blood levels of blood glucose (FBG), fasting insulin (FINS), C-peptide, non-esterified fatty acid (NEFA) TC, TG. This high-fat diet can be considered to be successful if the rat’s body weight becomes 20% higher than that of an average rat on the normal diet (Xu et al., 2015a). After 12 weeks, rats were euthanized and the spine was dissected to obtain several vertebral body-disk-vertebral body specimens. Several specimens from each rat were fixed in 4% paraformaldehyde for histological and immunohistochemical examination, and the others were frozen in liquid nitrogen for RNA extraction.

##### Immunohistochemistry

Specimens from high-fat diet group and normal diet group for histology were fixed with 4% paraformaldehyde at 4°C for 24 h, and decalcified using EDTA for 14 days. They were then sequentially dehydrated, embedded in paraffin, and sectioned at 5 μm. Sections were deparaffinized with xylene and rehydrated with graded ethanol. Endogenous peroxidase activity was blocked with 3% H_2_O_2_ for 10 min. Thin sections were incubated with trypsin for 20 min and incubated with 5% bovine serum albumin (BSA) and 1% Tween-20 in PBS for 30 min. to block unspecific antigens. Next, sections were incubated with antibodies against aggrecan, Col-II, MMP13, caspases 3, 7, and 9 (Abcam, Cambridge, MA, United States; 1:200), cleaved caspases 3, 7, 9 (Cell Signal Technology; 1:100), bcl-2 (Abcam;1:200) and PBS as negative controls overnight at 4°C. Sections were then incubated with corresponding HRP-conjugated secondary antibodies (Abcam; 1:5000) and counterstained with hematoxylin. Finally, five fields of view were chosen randomly from each section and imaged at 100 × [using Image J 1.48 software (Jiang et al., 2013)] in order to quantify cells positive for caspases 3, 7, and 9, and bcl-2. Cell numbers were calculated independently by three researchers. At least three thin sections were used from each specimen, and results averaged.

##### Tunel staining for apoptosis

After being deparaffinized with xylene and rehydrated with graded ethanol, thin sections were incubated with proteinase K (15 mg/ml) at 37°C for 15 min. A 3% solution of H_2_O_2_ was used for 5 min to quench endogenous peroxidase at room temperature, and sections were washed three times with phosphate-buffered saline (PBS). A cell death detection kit (Roche, Basel and Switzerland) was applied to the sections *in situ* according to the manufacturer’s protocols (Cho et al., 2015). In the fluorescence microscope, the wavelength ranges of excitation and emission were 450–500 nm and 515–565 nm, respectively (Jiang et al., 2013). Five fields were chosen randomly from each section (imaged at 100 ×) to quantify Tunel-positive cells, and at least three sections were used from each specimen.

#### Nucleus Pulposus Cell Culture

##### Cell extraction

IVDs were harvested from the lumbar spines of 12-week-old normal male Sprague–Dawley rats immediately after they were euthanized. The gel-like NP tissues of each group were separated from the disks, washed with Hank’s balanced salt solution (HANK Gibco, Grand Island, NY, United States) and cut into small fragments. Fragments were digested with 0.2% type II collagenase (Sigma, St. Louis, MO, United States) for 3 h, filtered through a cell strainer, and the isolated cells were rinsed twice with HANK. Cells were then cultured with complete culture medium (DMEM/F12, Gibco, Invitrogen, United States) containing 10% fetal bovine serum (FBS, Gibco, Invitrogen, United States) and antibiotics in a 5% CO_2_, 37°C environment. The medium was changed every 2–3 days (Diascro et al., 1998; Kong et al., 2014; Cheng et al., 2016).

##### Cell proliferation assay

Isolated NP cells were planted into 96-well plates (1 × 10^4^ cells per well) with complete culture medium for 12 h, and then cultured (as above) with palmitic acid (Sigma, Aldrich, United States), solubilized in 10% BSA solution for 48 h. The following concentrations of palmitic acid were used: 0, 50, 100, 150, 200, 400, 800, and 1600 μmol/L. NP cells in the culture medium were supplemented with Cell Counting Kit-8 (10 μL/100 μ, Sigma). After incubating for 2 h, cell density was estimated from the optical density measured at 450 nm (Wei et al., 2010).

##### Apoptosis quantification by flow cytometry

Apoptosis was quantified using the PE Annexin V apoptosis detection kit (BD Biosciences, San Diego, CA, United States) according to recommended protocols (Tints et al., 2014). NP cells were harvested as described above, collected together by centrifugation, washed with cold PBS twice, and then resuspended in 200 μL of 1 × annexin binding buffer at a concentration of 1 × 10^6^ cells per mL. A 200 μL sample of solution was treated with 5 μL of Annexin V-PE and 5 μL of 7-Amino-Actinnomycin (7-AAD) and incubated in the dark at room temperature for 30 min, followed by the addition of 400 μL of binding buffer. Stained cells were analyzed by a flow cytometer (EpicsAltra; Beckman Coulter, Fullerton, CA, United States). Annexin V-PE binding positive-staining cells were scored as apoptotic cells which were counted and represented as a percentage of the total cell count (Tints et al., 2014).

### Intracellular Measurement of Reactive Oxygen Species (ROS)

Intracellular ROS was evaluated by flow cytometry. This detects the oxidation of the intracellular fluorophore 2,7-Dichlorodi-hydrofluorescein diacetate (DCFH-DA) using Reactive Oxygen Assay Kit according to its protocols (Oksvold et al., 2002). The results are represented as average fluorescence intensity.

#### Real-time PCR

Total RNA was extracted from NP tissues or NP cells of each groups using TRIzol reagent (Invitrogen, Carlsbad, CA, United States). 1 μg of total RNA was used to synthesize cDNA (MBI Fermantas, Sankt Leon-Rot, Germany). For PCR amplification, 20 ml of reaction volume included 10 ml of 2 × SYBR Premix Ex Taq mixture (Takara, Shiga, Japan), 0.2 μmol/L each primer, 2 ml of twofold diluted cDNA and sterile distilled water according to the manufacturer’s protocols. Target genes included Aggrecan, Type II collagen (Col-II), Matrix metalloproteinase-3 (MMP-3), Matrix metalloproteinase-13 (MMP-13). The primer sequences used are shown in Table 1. The cycle threshold (Ct) values were collected and normalized to the housekeeping gene α-Tubulin. The 2^−ΔΔC_t_^ was calculated to estimate the relative mRNA levels of each target gene (Li et al., 2014b; Miao and Zhang, 2015; Terashima et al., 2016).

**TABLE 1:** Nucleic Acid Sequence of Forward (F) and Reverse (R) PCR Primers of Specific Genes.

**PCR Primers**	**Nucleic Acid Sequence**
Rat Aggrecan F	5′-GCAGCACAGACACTTCAGGA-3′
Rat Aggrecan R	5′-CCCACTTTCTACAGGCAAGC-3′
Rat Collegan-II F	5′-CTCAAGTCGCTGAACAACCA-3′
Rat Collegan-II R	5′-GTCTCCGCTCTTCCACTCTG-3′
Rat MMP-3 F	5′-TGATGAACGATGGACAGATGA-3′
Rat MMP-3 R	5′-AGCATTGGCTGAGTGAAAGAG-3′
Rat MMP-13 F	5′-CCTGGAGCCCTGATGTTTC-3′
Rat MMP-13 R	5′-TGGGTCACACTTCTCTGGTG-3′
Rat α-Tubulin F	5′-GAGCGCCCAACCTACACTAA-3′
Rat α-Tubulin R	5′-GGAAGTGGATGCGAGGGTAG-3′

#### Western blotting

Proteins were isolated using RIPA lysis buffer with protease inhibitors and phosphatase inhibitors (Beyotime, Nantong, China). The proteins were separated by SDS–PAGE gel electrophoresis and transferred to polivinyledene fluoride (PVDF) membranes. The PVDF membranes were cut according to different protein molecular weights and incubated with primary antibodies against pro PARP, pro caspase-3, 7, 9, cleaved PARP, cleaved caspase-3, 7, 9 (Cell Signal Technology, 1:1,000) and α-Tubulin (Cell Signal Technology, 1:1,000) and probed with the respective secondary antibodies. For MAPK pathway protein detection group, PVDF membranes were incubated with primary antibodies against phospho-p38, p38, phospho-ERK, ERK (Cell Signal Technology, 1:1,000) and α-Tubulin (Cell Signal Technology, 1:1,000), and then probed with the respective secondary antibodies. Blots were visualized using enhanced chemiluminescence reagents (Amersham Biosciences, Buckinghamshire, United States). Densitometry analysis was conducted with Image J (Bio-Rad, Hercules, CA, United States). This semi-quantitative assessment of proteins depends on the determination of gray levels, which was performed in Image J 1.48 (Elbaz et al., 2010; Li et al., 2014a,b).

### Statistical Analyses

Data are presented as means ± Standard Deviation (STD). All statistical procedures were carried out using SPSS 20.0 software (SPSS, Inc., Chicago, IL, United States). Interobserver reliability was analyzed using the kappa statistic (Olsen, 1989). Student’s *t*-test was used to evaluate differences between two group means, and ANOVA was used to compare means of three or more groups. The Wilcoxon rank sum test was used for non-normal data (as established by the one-Sample Kolmogorov–Smirnov Test (Bucova et al., 2015). The Chi-square test was used to compare proportions. Logistic regression was used to identify significant risk factors for disk degeneration. Differences were considered statistically significant if *p* < 0.05.

## Results

### Clinical Data

Clinical data are summarized in Table 2. Interobserver reliability of Pfirrmann grading was good (kappa values 0.722–0.789). All data except TG and Apo A was approximately Normal (*P* > 0.05) and there were no significant differences in sex proportion, height, or body weight between the “disk degeneration” and “non-degeneration” groups. However, the disk degeneration group were slightly older (*P* = 0.007) and had higher triglyceride levels (*P* = 0.012) and higher BMI (*P* < 0.001) than the normal group. Logistic regression results (Table 3) showed that serum triglyceride level (TG) was a significant predictor of disk degeneration. This was true even when data were adjusted for age, sex and BMI (Model 3) suggesting that TG may be an independent risk factor for IDD.

**TABLE 2:** Statistical table of influencing factors of intervertebral disk degeneration.

	**Non-degeneration group**	**Degeneration group**	***P*-value**
Sex	74	54	0.077
Male	47	26	
Female	27	28	
Age	61.1 ± 10.4	66.2 ± 10.3	0.007^∗∗^
Height (cm)	165.1 ± 7.3	162.6 ± 7.7	0.075
Weight (kg)	64.8 ± 9.7	68.1 ± 10.7	0.082
BMI	23.7 ± 2.9	25.7 ± 3.2	0.001^∗∗^
TG (mmol/L)	1.74 ± 0.98	2.45 ± 2.00	0.012^∗^
TC (mmol/L)	4.48 ± 1.01	4.53 ± 1.16	0.749
HDL (mmol/L)	1.01 ± 0.30	1.00 ± 0.27	0.83
LDL (mmol/L)	2.48 ± 0.74	2.48 ± 0.92	0.996
VLDL (mmol/L)	0.91 ± 0.54	1.08 ± 0.80	0.165
Apo A (g/L)	15.9 ± 15.3	19.55 ± 18.645	0.234

*Clinical data concerning intervertebral disk degeneration. BMI, Body Mass Index. TG, triglyceride. TC, Total Cholesterol. HDL, High Density Lipoprotein. LDL, Low Density Lipoprotein. VLDL, Very Low Density Lipoprotein. Apo A, Apolipoprotein. TG and Apo *A*-values are medians. Other values are means ± *SD*. ^∗^*P* < 0.05, ^∗∗^*P* < 0.01.*

**TABLE 3:** Odds ratios of triglyceride for intervertebral disk degeneration.

**Adjustment**	**Odds ratio**	**95% confidence interval**	***P*-value**
Model 1	1.410	1.060–1.876	0.018
Model 2	1.576	1.145–2.143	0.025
Model 3	1.554	1.129–2.139	0.037

*Bivariate logistic regression analysis showing how serum triglyceride level predicted intervertebral disk degeneration. CI, confidence interval. Model 1: unadjusted; Model 2: adjusted for age and sex; Model 3: adjusted for age, sex and BMI. ^∗^*P* < 0.05, ^∗∗^*P* < 0.01.*

### Intervertebral Disk Degeneration and Obesity in Rats

Baseline body weight of rats in the two diet groups were similar (Table 4). After 12 weeks, the high fat diet (HFD) rats were significantly heavier (*P* = 0.014), and had greater weight gain (*P* = 0.006). Average body weight was more than 20% greater in HFD rats than in ND rats, indicating that target levels of obesity were achieved. HFD rats also had higher serum levels of FBG (*P* = 0.046), FINS (*P* = 0.021), C-peptide (*P* = 0.016), NEFA (*P* = 0.009), TG (*P* = 0.004), and TC was non-significantly higher.

**TABLE 4:** Data for rats fed a high fat diet (HFD) compared with a normal diet (ND).

	**12 Weeks Old (Baseline)**			**24 Weeks Old**		
	**Obesity *n* = 10**	**Non-Obesity *n* = 10**	***P* value**	**Obesity *n* = 10**	**Non-obesity *n* = 10**	***P* value**
Weight (g)	220.61 ± 2.12	221.42 ± 2.95	0.113	412.2 ± 9.31	307.37 ± 14.31	0.014^∗^
Height (cm)	18.61 ± 2.12	18.52 ± 2.36	0.678	22.92 ± 4.14	21.71 ± 4.31	0.234
FBG (mmol/L)	4.76 ± 0.52	4.78 ± 0.61	0.102	7.18 ± 0.82	6.18 ± 0.75	0.046^∗^
FINS (μIU/mL)	13.023 ± 2.16	13.872 ± 2.31	0.325	13.715 ± 2.66	17.182 ± 3.47	0.021^∗^
C-peptide (μIU/mL)	1.28 ± 0.19	1.34 ± 0.26	0.325	1.39 ± 0.12	1.91 ± 0.31	0.016^∗^
NEFA (mmol/l)	239.88 ± 68.74	241.52 ± 80.48	0.624	259.37 ± 75.26	326.15 ± 79.75	0.009^∗∗^
TG (mmol/L)	0.42 ± 0.06	0.41 ± 0.05	0.624	0.93 ± 0.11	0.49 ± 0.04	0.004^∗∗^
TC (mmol/L)	1.41 ± 0.18	1.38 ± 0.14	0.093	1.91 ± 1.15	1.56 ± 0.25	0.062

*Obesity-related indices were generally higher for the HFD group. FBG, fast blood glucose. FINS, fasting insulin, NEFA, non-esterified fatty acid, TG, triglyceride. TC, Total Cholesterol. ^∗^*P* < 0.05, ^∗∗^*P* < 0.01.*

**FIGURE 1:**
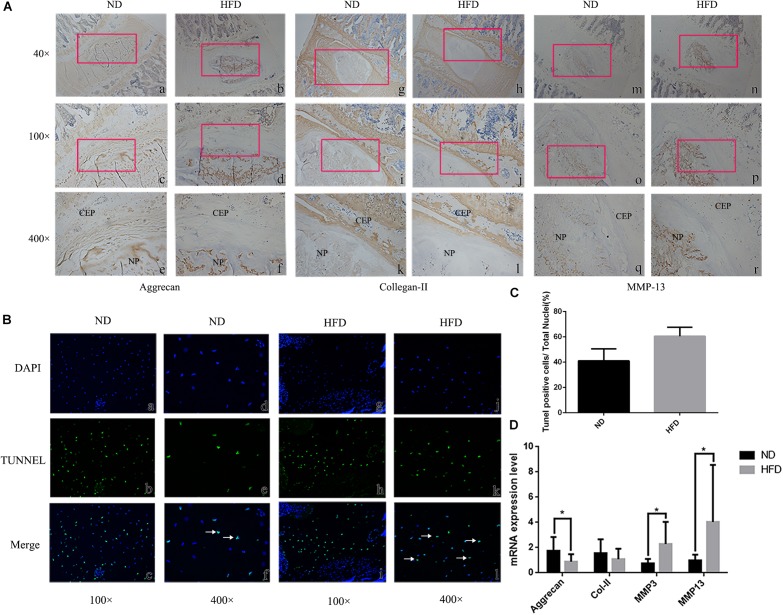
Intervertebral disc degeneration in rats fed a high fat diet (HFD) and normal diet (ND). **(A)** Immunohistochemistry at 40 ×, 100 ×, 400 × magnification, with location of enlarged regions indicated by red frames. NP = nucleus pulposus. CEP = cartilage endplate. **(B)** Tunel staining (b, e, h, k) showing apoptotic cells stained green. Blue fluorescence (a, d, g, j) represents DAPI-stained nuclei. In merged images (c, f, i, l) apoptotic cells are indicated by white arrows. **(C)** Statitical graph of Tunel staining positive cells. **(D)** Results of real-time PCR for Aggrecan, Collagen-II, MMP-3 and MMP-13 using RNA from nucleus pulposus tissue. ^∗^*P* < 0.05, ^∗∗^*P* < 0.01.

**FIGURE 2:**
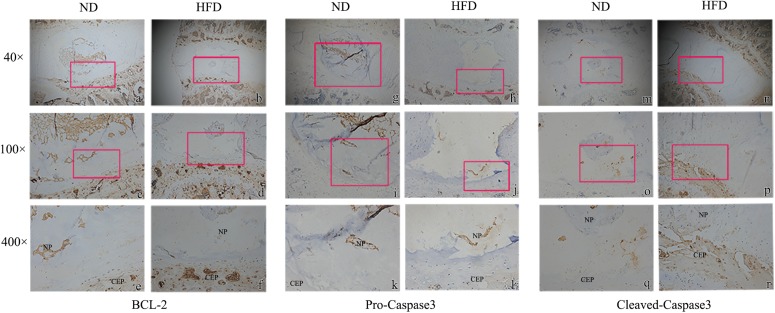
Immunohistochemistry of apoptosis markers with 40 ×, 100 ×, 400 × magnification. **(a–f)** Bcl-2; **(g–l)** pro-caspase 3; **(m–r)** cleaved-caspase 3. The enlarged regions are indicated by red frames. ^∗^ means *P*-value < 0.05, ^∗∗^ means *P*-value < 0.01.

Immunohistochemistry demonstrated that the extracellular matrix components aggrecan and Col-II were decreased in obese rats, whereas the matrix degrading enzyme MMP-13 was up-regulated significantly (Figure 1A). Tunel staining suggested that apoptosis developed significantly in the NP of obese rats though a few apoptotic cells were also observed in control rats (*P* < 0.05) (Figures 1B,C). In obese rats, the relative expression of anabolism genes aggrecan and Col-II were significantly down-regulated while the catabolism genes MMP-3 and MMP13 were significantly up-regulated (Figure 1D). Immunohistochemistry for detection of caspases 3 and 9 demonstrated that NP cells positive for cleaved caspases were more apparent in obese rats, and NP cells positive for pro caspases 3 and 9 were significantly reduced in obese rats. Bcl-2 was significantly decreased in obese rats, suggesting that apoptosis inhibition of Bcl-2 was decreased (Figure 2).

### Cell Culture Studies

**FIGURE 3:**
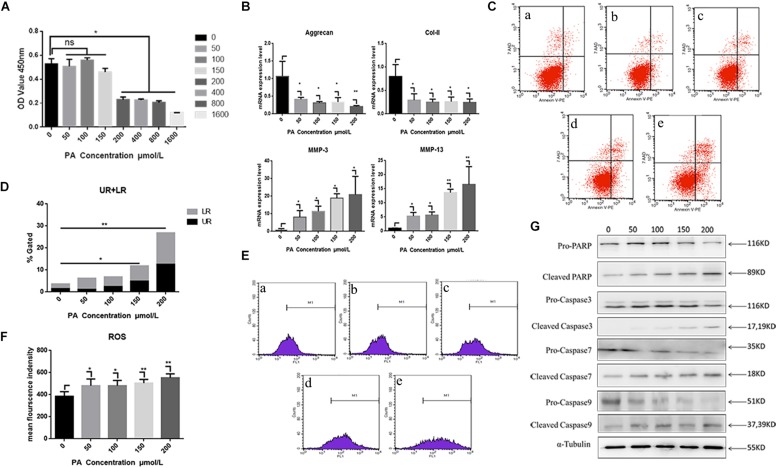
Nucleus pulposus cell degeneration and apoptosis detection. **(A)** Cell proliferation activity assay of different palmitic acid concentrations by Cell Count Kit-8. **(B)** The results of real-time PCR for Aggrecan, Collagen-II, MMP-3, and MMP-13 using RNA from nucleus pulposus cells initiated by different PA concentrations. **(C)** Apoptotic nucleus pulposus cells detection by flow cytometry. a:0 μmol/L, b:50 μmol/L, c:100 μmol/L, d:150 μmol/L, e:200 μmol/L. **(D)** Statistics analysis of apoptotic cells in upper right (UP) and low right (LR) regions. **(E)** Reactive oxygen species (ROS) detection by flow cytometry. a:0 μmol/L, b:50 μmol/L, c:100 μmol/L, d:150 μmol/L, e:200 μmol/L. **(F)** Statistics analysis of ROS represented by mean fluorescence intensity. **(G)** Western Blot of apoptosis related proteins in different PA concentration groups: pro-PARP, cleaved-PARP, pro-caspase3, cleaved-caspase3, pro-caspase7, cleaved-caspase7, pro-caspase9, and cleaved-caspase9. α-Tubulin is used as reference protein. ^∗^ means *P*-value < 0.05, ^∗∗^ means *P*-value < 0.01.

Exposure to the fatty palmitic acid (PA) generally inhibited viability and proliferation of NP cells. Proliferation was significantly reduced following 48 h exposure to PA at a concentration of 150 μmol/L, and this effect was increased at higher concentrations (*P* < 0.01) (Figure 3A). Therefore, we used a 150 μmol/L concentration of this fatty acid in subsequent experiments on cell proliferation. RT-PCR results demonstrated that PA treatment for 48 h induced a degenerative phenotype in NP cells: relative expressions of anabolism genes (Aggrecan, Col-II) were significantly down-regulated while the catabolism genes MMP-1, MMP13 were significantly up-regulated (Figure 3B). Flow cytometry showed that higher concentrations of PA (for 48 h) increased NP cell apoptosis, as indicated by Annexin V-PE binding (Figures 3C,D). Increases at 150 and 200 μmol/L concentration were significant (*P* < 0.05). Flow cytometry also showed that average fluorescence intensity of reactive oxygen species (ROS) increased significantly with concentrations of PA above 50 μmol/L (Figures 3E,F). Western blotting (Figure 3G) showed that exposure of NP cells to PA for 48 h down-regulated the apoptosis-related proteins pro-caspase 3, 7, 9, and pro-poly ADP-ribose polymerase (pro-PARP) in a dose-related manner. In contrast, concentrations of cleaved caspase 3, 7, 9 and cleaved PARP were significantly up-regulated.

### Activation of ERK Signal Pathway

**FIGURE 4:**
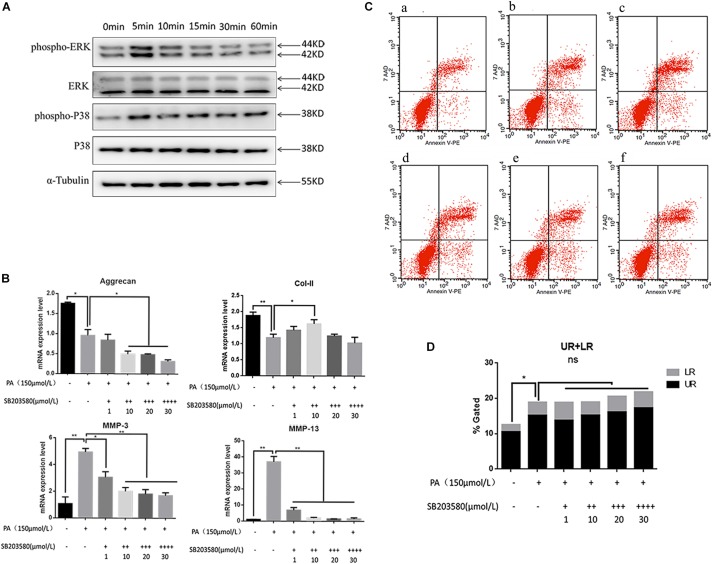
**(A)** Western Blot of MAPK pathway protein detection: P38 and ERK. **(B)** The results of real-time PCR for Aggrecan, Collagen-II, MMP-3, and MMP-13 using RNA from nucleus pulposus cells initiated by different P38 inhibitor (SB203580) concentrations and PA solution. **(C)** Apoptotic nucleus pulposus cells detection by flow cytometry. a:0 μmol/L PA + 0 μmol/L SB203580, b:150 μmol/L PA + 0 μmol/L SB203580, c:150 μmol/L PA + 1 μmol/L SB203580, d:150 μmol/L PA + 10 μmol/L SB203580, e:150 μmol/L PA + 20 μmol/L SB203580, f:150 μmol/L PA + 30 μmol/L SB203580. **(D)** Statistics analysis of apoptotic cells in upper right (UP) and low right (LR) regions.

**FIGURE 5:**
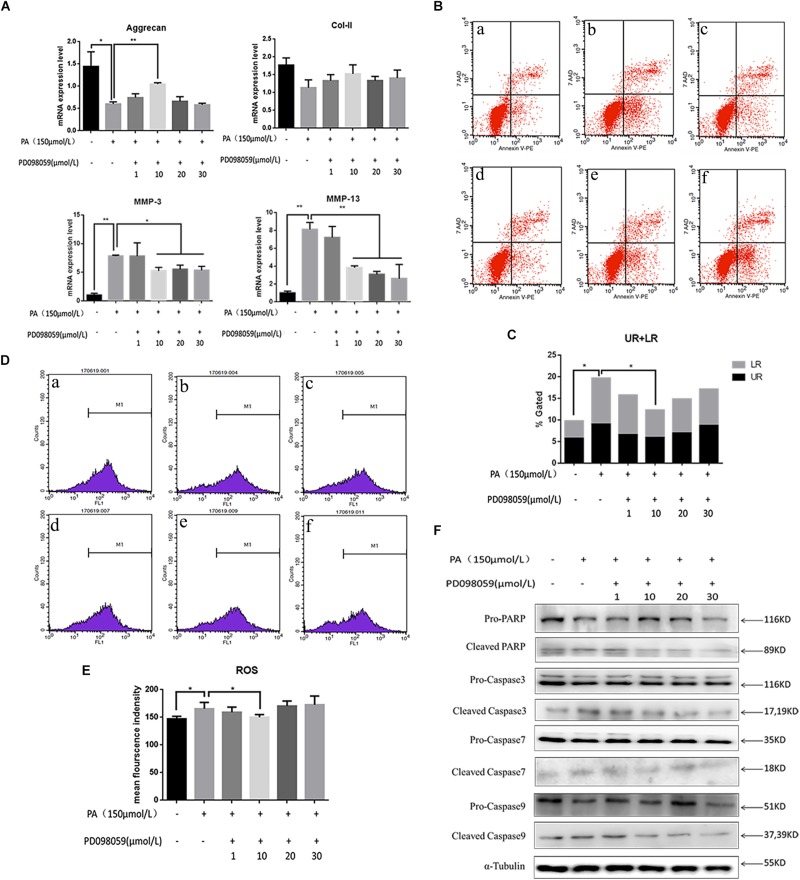
**(A)** The results of real-time PCR for Aggrecan, Collagen-II, MMP-3, and MMP-13 using RNA from nucleus pulposus cells initiated by different ERK inhibitor (PD098059) concentrations and PA solution. **(B)** Apoptotic nucleus pulposus cells detection by flow cytometry. a:0 μmol/L PA + 0 μmol/L PD098059, b:150 μmol/L PA + 0 μmol/L PD098059, c:150 μmol/L PA + 1 μmol/L PD098059, d:150 μmol/L PA + 10 μmol/L PD098059, e:150 μmol/L PA + 20 μmol/L PD098059, f:150 μmol/L PA + 30 μmol/L PD098059. **(C)** Statistics analysis of apoptotic cells in upper right (UP) and low right (LR) regions. **(D)** Reactive oxygen species (ROS) detection by flow cytometry. a:0 μmol/L PA + 0 μmol/L PD098059, b:150 μmol/L PA + 0 μmol/L PD098059, c:150 μmol/L PA + 1 μmol/L PD098059, d:150 μmol/L PA + 10 μmol/L PD098059, e:150 μmol/L PA + 20 μmol/L PD098059, f:150 μmol/L PA + 30 μmol/L PD098059. **(E)** Statistical analysis of ROS represented by mean fluorescence intensity. **(F)** Western Blot of apoptosis related proteins in different P38 inhibitor (PD098059) concentrations and PA solution group: pro-PARP, cleaved-PARP, pro-caspase3, cleaved-caspase3, pro-caspase7, cleaved-caspase7, pro-caspase9, and cleaved-caspase9. α-Tubulin is used as reference protein.

Involvement of the MAPK signaling pathway is very common in apoptosis. In the present results, the phospho-ERK and phospho-P38 proteins were significantly up-regulated 5 min after PA stimulation, and began to reduce after 10 min, suggesting that the fatty acid can activate the MAPK pathway in NP cells (Figure 4A). The relative quantity of phospho-ERK/ERK and phospho-P38/P38 was significantly higher in 5 min (*P* < 0.05). When NP cells were pretreated with P38 inhibition (SB203580) for 2 h and stimulated by PA for 48 h, the expression of aggrecan and Col-II could be rescued while MMP-3, MMP-13 were inhibited (Figure 4B). However, the apoptosis could not be inhibited by P38 inhibitions as showed in the flow cytometry (Figures 4C,D). When NP cells were pretreated with ERK inhibition (PD098059) for 2 h and stimulated by PA for 48h, there appeared to be narrow differences in the expression of all the transcriptions (Aggrecan, Col-2, MMP-3, MMP-13) which suggested PA could induce NP degeneration via ERK signal pathway (Figure 5A). Meanwhile, Apoptosis could be inhibited by ERK inhibition as the flow cytometry results showed (Figures 5B,C). Average fluorescence intensity of ROS was also significantly decreased using ERK inhibition (Figures 5D,E). When NP cells were pretreated with ERK inhibition, the apoptosis-related protein was changed, respectively. With the concentrations of ERK inhibition increasing, the pro-caspase3, 7, 9 and pro-PARP were up-regulated while cleaved-caspase3, 7, 9 and cleaved–PARP were down-regulated (Figure 5F), which suggested that apoptosis induced by PA was via ERK signal pathway, rather than P38 pathway, though both of they might be the pathway of intervertebral disk degeneration.

## Discussion

### Summary of Results

The clinical study confirmed known associations between IDD and obesity, and showed for the first time that high serum levels of lipids (in particular triglyceride) are an independent risk factor for IDD, even after correction for BMI and age. The animal model confirmed that a high fat diet can lead to obesity and to high serum levels of lipids, including triglyceride. The cell culture studies showed that nucleus pulposus cells exposed to high levels of a fatty acid (palmitic acid) exhibit decreased markers of anabolism, increased markers of catabolism, increased levels of apoptosis, and increased activation of caspases 3, 7, 9, and PARP. Cell signaling following PA stimulation was primarily via the MAPK signal pathway, especially the ERK pathway was involved in NP cell apoptosis.

### Strengths and Weaknesses of the Study

Our clinical study was cross-sectional and could only demonstrate associations between specific risk factors and IDD, rather than a causal link. Nevertheless, results from all three studies were consistent and give a plausible explanation for how obesity might cause IDD by influencing the concentration of certain lipids in the blood, which then impair the metabolism of cells in the disk nucleus pulposus. The clinical study used bodyweight and BMI to represent all biomechanical influences in IDD, and it is possible that more specific ergonomic-related risk factors might have had a greater influence. Much of the work involved a rat model of IDD, and small animal models of IDD have some weaknesses (Alini et al., 2008). However, the major weaknesses (that laboratory animal disks are too small and young to experience disk metabolite transport problems, or vulnerability to mechanical failure) have little relevance to the experiments reported here, so our results are probably a reliable guide to human disks. The cell culture experiments used palmitic acid to represent all blood lipids that are elevated in obesity (including triglyceride) but palmitic acid has been used before in similar studies (Drosatos-Tampakaki et al., 2014) and there is little reason to suppose that other lipids would have a markedly different effect. When used in high concentration, DMSO, the inhibitors solvent of ERK and P38 pathway itself may have a cytotoxic effect on the NP cells which might influence our apoptosis results. And our study has not identified a specific molecular mechanism linking NP cell apoptosis with IDD via the ERK pathway, but we aim to explore this possibility in future research.

### Relationship to Previous Work

As described in the Introduction, many previous studies have examined the relationship between obesity and IDD, but few have considered both biomechanical and metabolic factors. Obese patients are not only heavier, but often exhibit hypertriglyceridemia, and it possible that high blood lipid levels could impair disk cell metabolism and influence inflammation. However, specific mechanisms linking blood lipids to IDD have not previously been reported. Results presented here are in broad agreement with previous work cited in the section “Introduction.” The involvement of MAPK pathway in apoptosis is very common. Phospho-P38 and phospho-ERK was activated in the 5 min and then reduced which suggests degeneration and apoptosis may via MAPK signal pathway. What’s more, PA with ERK inhibition (PD098059) could inhibit degeneration and apoptosis while PA with P38 inhibition (SB203580) could only inhibit degeneration. This suggest that the ERK pathway plays an important role in NP cell apoptosis rather than P38 initiated by PA solution though they could both lead to degeneration especially in disk catabolism. Other studies have reported that diabetes or high glucose can also cause IDD. However, the serum glucose level of our obesity rats was also higher than the normal group, which could not be excluded in our study.

### Interpretation of Results

Obesity appears to influence disk degeneration by a direct biochemical influence of fatty acids on disk cell metabolism. Palmitic acid solution was shown to induce catabolic degenerative changes and apoptosis in NP cells. It was also shown to upregulate the apoptosis-related proteins caspase 3, 7, 9, and PARP. Mechanical factors associated with obesity (such as increased body weight) may play an additional role, but the present results showed (in humans) that the influence of blood lipids can be independent of associated mechanical factors. This was confirmed in the rat model also. It should be noted that mechanical influences in IDD will be less in a horizontal quadruped such as a rat compared to an upright bipedal human. However, the present results do not rule out the possibility of additional direct mechanical influences in the degeneration of human disks.

### Clinical Relevance

Results of this study suggest that clinical interventions to reduce hypertriglyceridemia could have additional benefit for the intervertebral disks.

## Data Availability Statement

All datasets generated for this study are included in the manuscript/supplementary files.

## Ethics Statement

The studies involving human participants were reviewed and approved by Ethics review committee of Sir Run Run Shaw Hospital. The patients/participants provided their written informed consent to participate in this study. The animal study was reviewed and approved by the Animal Care and Use Committee at Zhejiang University. Written informed consent was obtained from the individual(s) for the publication of any potentially identifiable images or data included in this article.

## Author Contributions

All authors were involved in conception and design and take responsibility for the integrity of the data analysis. XZ, JC, SF, and FZ designed the study. BH, JW, ZS, and JL conducted the study. JL, YC, and SL collected and analyzed the data. YC, SL, SF, and FZ interpreted the data. XZ, JC, BH, and FZ drafted the manuscript.

## Conflict of Interest

The authors declare that the research was conducted in the absence of any commercial or financial relationships that could be construed as a potential conflict of interest.
